# On Physiognomy

**Published:** 1919-06-21

**Authors:** 


### ON PHYSIOGNOMY.

### By Caduceus.

"Mary Queen of Scots wears surgical boots and is subject to fits, near the Horse Shoe in Tottenham Court Road."

THIS is not what it looks to be, a further item of asylum news, but on the contrary one of those delightful sallies that the Victorians, more solemn but greater than ourselves, seemed unable to taste. One does it no sort of justice, however, thus lifting it from its setting. Samuel Butler is on the subject of facial resemblances and maintains that the very images of famous people may be seen often enough, if you get about a little. Here, of course, the likeness is only skin deep; but I believe there are complete, thorough-going resemblances to be found, detailed close similarity in face and character both. If it were not for the shortness of human life one would come upon this phenomenon more frequently. Just consider the obstacles in the way of the observer. Often enough you see in the tube or at a public gathering some one with the very same type and cut of face as an acquaintance, but the keenest investigator could hardly go up to him, still less to her, and enter into conversation on the strength of that. Even given the requisite nerve (and the requisite complaisance) mere conversation would not reveal nearly enough. Further, the interval of time between the two meetings may have been too great, so that you have forgotten all about the first before you meet his counterpart. Or his display of character may not have been striking, so that you have not got him well in mind. Still one does find instances in point now and again; and here are two, both of some medical interest.

In a ship returning from the Antipodes in the 'nineties was a Queensland planter, who got drunk every night. He was a man about five feet nine in height and though not fat, he was the reverse of bony. The intervals between neck and shoulder, between waist and axilla, were well rounded off. His hair was dark and smooth, he had a big moustache, rather drooping. It was a wide face with round eyes, each orbit directed a trifle away from the other. The nose was a good one, although concave rather than straight. But the general impression was of likeness to a seal. Dickens would have put that in the forefront of his description. Well, I had forgotten all about this jolly performer, all about the distended elastic round his weekly bundle of wine cards, until the other day, when I saw a general practitioner's medical certificate that a certain potential recruit suffered from palpitation and dyspepsia, the result of chronic alcoholism. For that recruit was the type, the very type.

To begin with he looked prosperous—and trustworthy: an hotel proprietor might have cashed a cheque for him. And there was the same suggestion of a seal. The eyes were a different colour, but they were a little goggly just the same. The hair was thinner, but that made no matter. The stature was a little less and the body had run a little more to seed, but there was the same natural "stream line" about its well developed superficial fascia; the very same marine suggestion about the moustache. No mistake about it, he was the type; a born drunkard from his mother's womb.

That may well give the impression of over-confident generalisation; but bear with me while I relate, more shortly, the second instance.

Scene: a certain familiar red brick building on the Embankment. Persons; an examiner and his examinee. Subject of conversation: an affection of the spleen. I had said so-and-so about the diseased organ, and when pressed had appealed to the authority of the text-books. My appeal was allowed; but, said Rhadamanthus, rising and striking with face and figure an attitude, "Is not the profession saying with a louder and ever louder voice—not so-and-so but such-and-such?" This display of rhetoric struck me, even in that preoccupied mood, as rather odd, from one in his position to one in mine—so wax-to-receive, so little likely to encroach as I was! But ingenuous youth knows little of the ways of the world. A few years later, in the column of the medical journals which is more read than any others, I saw an account of his career. It appeared that before he had got much practice he would frequent Alpine hotels and mix with the guests dressed in frock coat and top hat, even making some of the smaller ascents thus professionally accoutred. Reflecting that obituary notices are studies in the understatement of censure, I began to place him. But I had not yet begun to connect this inward and spiritual grace with his particular outward and visible form. He was physically a big man all over—a big square head, big arms, big chest, big thighs, big legs, Eyes very blue, mock stedfast; skin clear and healthy; a firm mouth; the expression one of resolute pretension; the general suggestion as of a tough conscience, a cool head and a good digestion.

Now some years later again, I began to notice that the patients of one general practitioner used often to get from him envelopes, having his name and address printed in very bold type, in which rather superfluously to reply to him. None of the other doctors seemed to care about so much correspondence. One day I saw him—here was Rhadamanthus all over again! The same big imposing façade, just a little "stuccoasque"; with the usual small exceptions to prove the rule. The upper lip was as stiff, but the blue eyes in the healthy face were quieter and the head was not carried so far back (he had not written a standard if hardly erudite hand-book on therapeutics). He was younger, too, and the poll, though as irritatingly square, was not as shiny. His talk was well in character, however; and I am now looking out, not only for another bibulous seal, but also for another big *bluffeur*. My faith in determinism is so unfashionably lively that I should not wonder if I found both before long.

